# Two yellow luminescence bands in undoped GaN

**DOI:** 10.1038/s41598-018-26354-z

**Published:** 2018-05-25

**Authors:** M. A. Reshchikov, J. D. McNamara, H. Helava, A. Usikov, Yu. Makarov

**Affiliations:** 1 Department of Physics, Virginia Commonwealth University, Richmond, VA 23284 USA; 2 Nitride Crystals, Inc., 9702 Gayton Rd., Richmond, VA 23238 USA; 3 Saint-Petersburg National Research University of Information Technologies, Mechanics and Optics, 49 Kronverkskiy Ave., 197101 Saint Petersburg, Russia

## Abstract

Two yellow luminescence bands related to different defects have been revealed in undoped GaN grown by hydride vapor phase epitaxy (HVPE). One of them, labeled YL1, has the zero-phonon line (ZPL) at 2.57 eV and the band maximum at 2.20 eV at low temperature. This luminescence band is the ubiquitous yellow band observed in GaN grown by metalorganic chemical vapor deposition, either undoped (but containing carbon with high concentration) or doped with Si. Another yellow band, labeled YL3, has the ZPL at 2.36 eV and the band maximum at 2.09 eV. Previously, the ZPL and fine structure of this band were erroneously attributed to the red luminescence band. Both the YL1 and YL3 bands show phonon-related fine structure at the high-energy side, which is caused by strong electron-phonon coupling involving the LO and pseudo-local phonon modes. The shapes of the bands are described with a one-dimensional configuration coordinate model, and the Huang-Rhys factors are found. Possible origins of the defect-related luminescence bands are discussed.

## Introduction

Point defects play a significant role in the electrical and optical properties of GaN semiconductor and GaN-based electronic and photonic devices. However, a majority of the point defects in this semiconductor remain unidentified. In particular, researchers have debated for several decades about the origin of the notorious yellow luminescence (YL) band, which has a maximum at about 2.2 eV. The YL band is most often attributed to either gallium vacancy (V_Ga_)-related defects^1–3^ or carbon-related defects^4–6^. Although early first-principles calculations strongly favored the V_Ga_ defects or the V_Ga_O_N_ complexes to be responsible for the YL band^7,8^, recent calculations predict that it is rather caused by an isolated carbon defect, C_N_^9,10^ or the C_N_O_N_ complex^11,12^. As for the V_Ga_-related defects, only the V_Ga_O_N_-2H and V_Ga_-3H complexes can contribute to the YL band in *n*-type GaN according to recent calculations^13^.

A very popular viewpoint is that several types of defects may cause luminescence bands with similar positions and shapes, so that it may be difficult to distinguish them in the experimentally observed broad YL band. In particular, Armitage *et al*.^14^ suggested that a strong YL band in C-doped GaN (with [C] = 2 × 10^18^ cm^−3^ and [V_Ga_] < 1 × 10^16^ cm^−3^) is due to C, and a strong YL band in undoped GaN ([C] = 6 × 10^16^ cm^−3^ and [V_Ga_] > 1 × 10^17^ cm^−3^) is due to V_Ga_. No difference in positions and shapes of the broad YL bands was reported in this work, yet the activation energies, *E*_*A*_, obtained from the thermal quenching of the YL band, were found to be different: *E*_*A*_ = 1.04 eV and 0.64 eV for the “C-rich” and “V_Ga_-rich” GaN samples, respectively. Note, however, that the activation energies obtained from photoluminescence (PL) quenching may vary for different samples and are not necessarily equal to the defect ionization energy^15^.

Both positive and negative correlations between the intensity of the YL band and the concentrations of the C and V_Ga_ defects were reported. For example, Xu *et al*.^16^ observed a much stronger YL band in high-resistivity GaN with high concentration of C and very low concentration of V_Ga_ as compared to conductive *n*-type GaN samples. In the latter, the intensity of the YL band correlated with the concentration of V_Ga_ in three samples. On the other hand, Huber *et al*.^17^ observed that the YL intensity increases linearly with increasing concentration of C from 1.3 × 10^16^ to 1.24 × 10^17^ cm^−3^ and it decreases with increasing concentration of V_Ga_ from 8 × 10^16^ to 6 × 10^17^ cm^−3^ in the same set of four GaN samples.

It is difficult to verify or reject the assumption that the YL band is caused by different types of defects when the band is broad and featureless. However, recently we have discovered the zero-phonon line (ZPL) and phonon-related fine structure of a distinct YL band, and have thus labeled it YL1^18^. The ZPLs at 2.57 eV (at 18 K) and 2.59 eV (at 50 K) were attributed to no-phonon transitions of electrons from shallow donors (18 K) and from the conduction band (50 K) to a deep defect with the thermodynamic charge transition level at 0.916 ± 0.003 eV above the valence band. The intensity of the ZPL is 1% of the intensity of the YL1 band maximum.

The YL1 band with above parameters was initially observed in eight undoped GaN samples grown by hydride vapor phase epitaxy (HVPE), one undoped and three Si-doped GaN samples grown by metalorganic chemical vapor deposition (MOCVD)^18^. Since then, we have identified the YL1 band with its ZPL and fine structure in other samples, including C-doped and Fe-doped semi-insulating GaN layers on sapphire grown by MOCVD, Fe-doped, freestanding GaN grown by HVPE, and undoped GaN grown by molecular beam epitaxy (MBE). The yellow bands with different thermal quenching behavior in most cases were identified as the same YL1 band^19^. These facts indicate that only one type of defect causes the YL1 band in majority of GaN samples, grown by different techniques and exhibiting various electrical conductivity behavior. The determined electron-capture coefficient (*C*_*nA*_ = 1.1 × 10^−13^ cm^3^/s) and hole-capture coefficient (*C*_*pA*_ = 3 × 10^−7^ cm^3^/s) for the YL1 band also serve as its fingerprints and help to recognize it in samples where no fine structure can be observed.

Earlier we reported on another PL band with well-resolved ZPL and fine structure^20^. In low-temperature steady-state PL (SSPL) spectra, a strong “red” band with a maximum at 1.8 eV, called hereafter the RL3 band, is observed in select HVPE GaN samples^20^. The band has an unusual shape, with a shoulder at the high-energy side. A very similar PL band with a maximum at 1.8 eV in GaN grown by HVPE and containing high concentrations of C and O (about 10^19^ cm^−3^) was reported by other researchers^21,22^. The ZPL at 2.36 eV was associated with the RL3 band in ref.^20^, because its intensity relative to the RL3 band maximum (about 10%) was the same in several GaN samples grown by HVPE, and it was not possible to resolve any other band in this spectral region by changing temperature or excitation intensity in SSPL measurements.

In this work, we provide evidence that the ZPL and phonon-related fine structure attributed earlier to the RL3 band, in fact belong to a new YL band (labeled YL3). The YL3 band can be clearly separated from the RL3 band by using the time-resolved PL (TRPL) technique due to very different PL lifetimes of the RL3 and YL3 bands.

## Results

### Steady-state photoluminescence spectra

**Figure 1:**
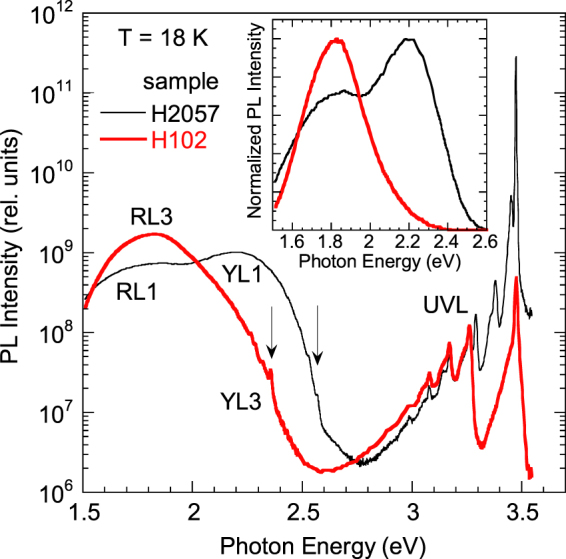
The SSPL spectra from GaN samples: group I (H2057) and group II (H102). *T* = 18 K and *P*_*exc*_ = 0.0002 W/cm^2^. The arrows show ZPLs at 2.36 and 2.57 eV. The inset shows normalized PL spectra in linear scale.

Low-temperature SSPL spectra from two representative samples are shown in Fig. 1. As it is explained in the Methods section, samples were divided into two groups according to their distinctive PL features. For group I samples (H2057 in Fig. 1), the broad band between 1.5 and 2.6 eV consists of the RL1 and YL1 bands^18,23^. The YL1 band has a maximum at 2.20 eV and the ZPL at 2.57 eV at low temperatures, and the RL1 band has a maximum at 1.80 eV. The RL1 band can be recognized by a slow decay after a laser pulse in TRPL, slower than that for the YL1 band. For example, the PL lifetimes for the YL1 and RL1 bands in sample H2057 at 100 K are 290 and 720 µs, respectively.

The lifetimes of the YL1 and RL1 bands are inversely proportional to the concentration of free electrons, which indicates that the bands are caused by transitions of electrons from the conduction band to two deep-level defects at *T* = 100 K^24^. At temperatures below 50 K, the decays of the YL1 and RL1 bands are nonexponential and close to a power dependence, which is typical for donor-acceptor-pair (DAP)-type transitions^25^. A sharp peak at 3.474 eV in the SSPL spectrum from sample H2057 is identified as an exciton bound to a neutral shallow donor. The blue shift by 3 meV as compared to the exciton position in bulk GaN is caused by small in-plain biaxial strain in the 24 μm-thick GaN layer grown on sapphire substrate^3,26^. The exciton emission is very strong, and its intensity increases linearly with excitation intensity for group I samples.

In samples from group II (H102 in Fig. 1), the RL3 band with a maximum at 1.80 eV is the strongest defect-related feature. The PL spectra from samples of group II were identical, and the RL3 band could not be resolved into separate bands in the SSPL spectra by changing temperature or excitation intensity^20^. Although the red bands from samples of groups I and II have the same position, the RL3 band can be distinguished from the RL1 band by a very fast, exponential decay after a laser pulse, with a characteristic lifetime of 15 ns at temperatures between 18 and 200 K. Another distinction is that the RL3 band has an asymmetric shape, with less steep high-energy side^20–22^. A sharp line at 2.36 eV was erroneously identified as the ZPL of the RL3 band in ref.^20^. The RL1 and RL3 bands should not be confused with other defect-related PL bands in this part of the spectrum. In particular, the RL2 band with a maximum at ~1.8 eV in undoped, semi-insulating, Ga-rich GaN is attributed to an internal transition within an unknown defect. The RL2 band is characterized with a very long exponential decay after a laser pulse, with a characteristic lifetime of 110 μs at *T* = 15 K^3^. Note that red bands related to some deep defects are also observed in GaN heavily doped with Mg^3,27^.

The exciton emission from group II samples (the donor-bound exciton at 3.474 eV for sample H102) is much weaker at low *P*_exc_ than that for group I samples. More importantly, at low excitation intensities, it increases super-linearly with the excitation intensity, as (*P*_exc_)^*n*^, with *n* ≈ 1.5 both at 18 K and 300 K. Such behavior is typical for high-resistivity samples, where both free electrons and holes are in deficit. In particular, the exciton emission in high-resistivity, Zn-doped GaN samples grown by HVPE also exhibited the (*P*_exc_)^1.5^ power dependence^28^.

For all HVPE GaN samples, a weak UVL band with the main peak at 3.26 eV followed by a few LO phonon replicas is observed at 18 K. This band is quenched above 100 K, so that a very weak, Zn-related BL1 band can be detected at 2.9 eV in the temperature range of 160–220 K. Out of 30 HVPE GaN samples, ten samples clearly belong to group I, and four samples belong to group II. In some other HVPE GaN samples (including sample RS280 analyzed in ref.^12^), both RL1 and RL3 bands contributed nearly equally to the red band in SSPL. Note that in MOCVD GaN (such as sample cvd3540), only the YL1 band was observed at these photon energies^18^.

### Evolution of photoluminescence spectra after a laser pulse

**Figure 2:**
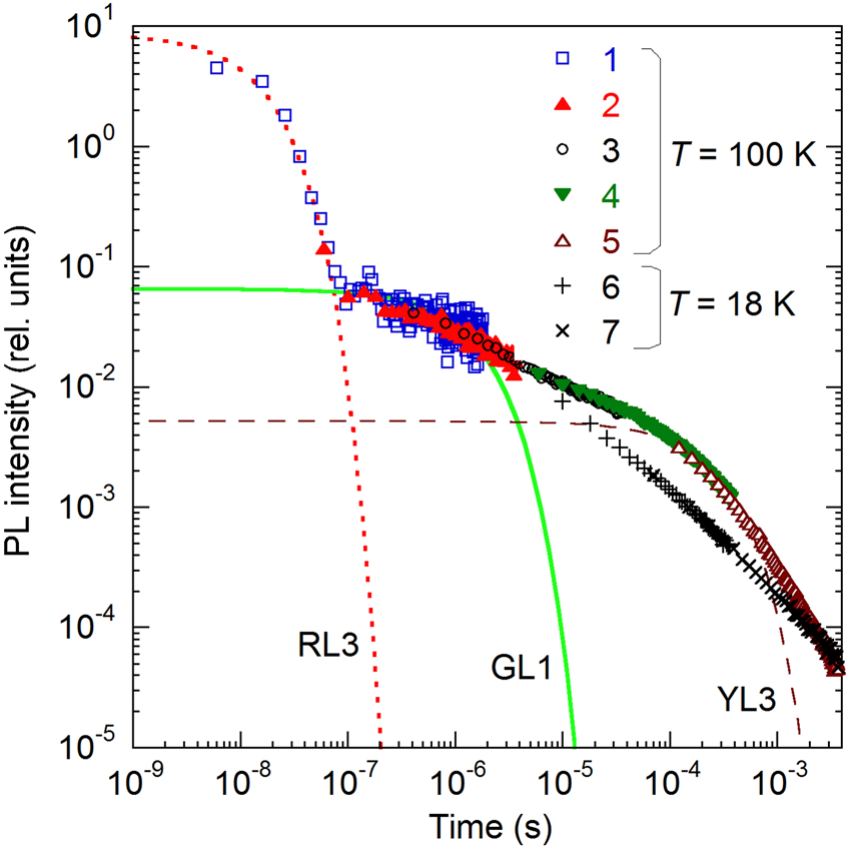
The PL transients at 2.1 eV from sample H106 (group II). The symbols show every 100^th^ point. At 100 K, the PL decays are formed by five overlapped transients taken with different time scales of the oscilloscope. At 18 K, only slow transients are shown for clarity, because at shorter times the PL decay was nearly identical to that at 100 K. The dotted, solid, and dashed lines are calculated using Eq. (1) with the parameter *τ* equal to the following: 15 ns (RL3), 1.5 μs (GL1), and 270 μs (YL3), respectively.

Examples of PL decays, *I*^*PL*^(*t*), at a selected photon energy ($\hslash \omega =2.1$ eV) are shown in Fig. 2.

For PL caused by transitions of electrons from the conduction band to a defect level, the PL decay after the laser pulse is expected to be exponential, with a characteristic PL lifetime, *τ*, which can be found by fitting the experimental data with the following expression:

1 $${I}^{PL}(t)={I}^{PL}(0)\,\exp (\,-\,\frac{t}{\tau }).$$

**Figure 3:**
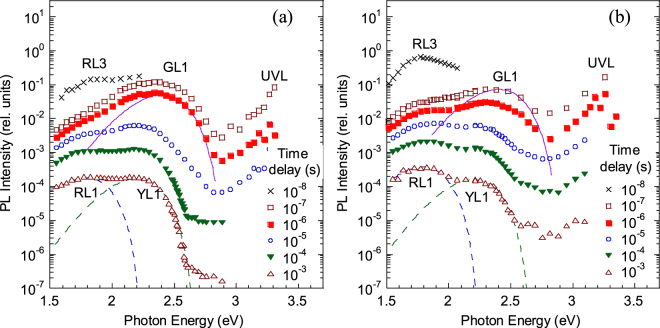
The TRPL spectra for group I samples at different time delays (from 10^−8^ to 10^−3^ s) at *T* = 18 K. (**a**) Sample H2057, (**b**) sample H201. The lines are calculated using Eq. (2) with the following parameters: *S*_*e*_ = 8.5, *E*_0_^*^ = 2.93 eV, $\hslash {\omega }_{{\rm{\max }}}=2.40$ eV (for the GL1 band), *S*_*e*_ = 7.4, *E*_0_^*^ = 2.66 eV, $\hslash {\omega }_{{\rm{\max }}}=2.20$ eV (for the YL1 band), and *S*_*e*_ = 6.2, *E*_0_^*^ = 2.22 eV, and $\hslash {\omega }_{{\rm{\max }}}=1.78$ eV (for the RL1 band).

**Figure 4:**
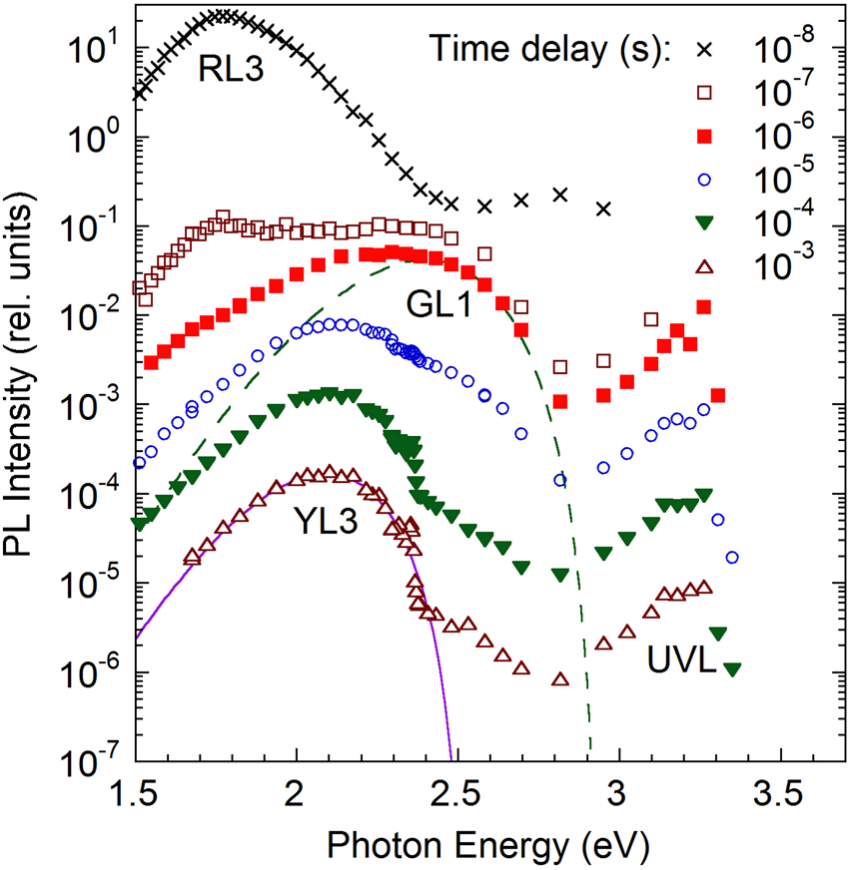
The TRPL spectra for group II sample H106 at different time delays (from 10^−8^ to 10^−3^ s) at *T* = 18 K. The lines are calculated using Eq. (2) with the following parameters: *S*_*e*_ = 8.5, *E*_0_^*^ = 2.93 eV, $\hslash {\omega }_{{\rm{\max }}}=2.40$ eV (for the GL1 band) and *S*_*e*_ = 7.0, *E*_0_^*^ = 2.52 eV, $\hslash {\omega }_{{\rm{\max }}}=2.09$ eV (for the YL3 band).

In Fig. 2, we can distinguish more than one exponential component, which indicates that different defect-related bands dominate at different time delays for a selected $\hslash \omega$. Nonexponential decay of PL (often the *t*^−*m*^ dependence with *m* ≈ 1) is usually a signature of DAP transitions that are common at very low temperatures^25^. In this case, the PL lifetime cannot be determined. From PL decays at different $\hslash \omega$, the TRPL spectra can be reconstructed for different time delays (Figs 3 and 4).

To resolve the PL bands in these spectra, we used the following expression for a PL band shape obtained using a one-dimensional configuration coordinate model^29^:

2 $${I}^{PL}(\hslash \omega )={I}_{max}^{PL}\exp [\,-\,2{S}_{e}{(\sqrt{\frac{{{E}_{0}}^{\ast }-\hslash \omega }{{{E}_{0}}^{\ast }-\hslash {\omega }_{max}}}-1)}^{2}].$$

Here, ${I}_{{\rm{\max }}}^{PL}$ is the PL intensity at the band maximum, *S*_*e*_ is the Huang-Rhys factor for the excited state, $\hslash \omega$ and $\hslash {\omega }_{{\rm{\max }}}$ are the photon energy and position of the band maximum, respectively, ${{E}_{0}}^{\ast }={E}_{0}+0.5\hslash {\rm{\Omega }}$, ${E}_{0}$ is the ZPL energy, and $\hslash {\rm{\Omega }}$ is the energy of the dominant phonon mode in the excited state. We have found that the shape of a particular defect-related band, such as the YL1 band, is identical in different samples and the same in SSPL and TRPL measurements^18^. Thus, Eq. (2) helps us to reliably resolve overlapped PL bands. All the parameters (except for the arbitrary ${I}_{{\rm{\max }}}^{PL}$) were taken from the fits of defect-related band shapes in conditions where the contributions from other defects were negligible.

At short time delays (*t* ≈ 10 ns), the RL3 band was detected in almost all of the HVPE GaN samples (Figs 3 and 4). Note that the PL intensity in Figs 2–4 is shown in the same relative units, and the samples were measured under identical conditions. The decay of the RL3 band is exponential, with *τ* ≈ 15 ns, at temperatures between 18 and 200 K. The exponential decay at very low temperatures and the temperature independence of the PL lifetime indicate that the related transition is internal; i.e., the transition occurs between two states of the same defect located in the band gap. The RL3 band is very strong in group II samples (Fig. 4). In group I samples, it is much weaker (Fig. 3), but still can be detected due to the characteristic fast and exponential decay. In fact, we have found the RL3 band in nearly all HVPE-grown GaN samples, including freestanding GaN templates from the Samsung Advanced Institute of Technology which were thoroughly studied by several research groups^3,30,31^. Interestingly, we never observed the fast RL3 band in GaN samples grown by MOCVD or MBE.

At longer time delays (*t* ≈ 0.1–1 µs), the GL1 band with a maximum at 2.40 eV can be well resolved in all HVPE GaN samples (it is absent in MOCVD and MBE GaN). The GL1 band decays exponentially in time with a characteristic lifetime of about 2 µs at 18 K. This band is attributed to the so-called “giant trap”, which introduces two transition charge levels (−/0 and 0/+) and a ladder of excited states near the conduction band of GaN when the defect is positively charged^32^.

After the GL1 band decays, the yellow and red bands appear at longer time delays. The slow red band, which is the RL1 band, is observed only for HVPE GaN samples of group I (Fig. 3). The group I samples also reveal the usual YL1 band with a maximum at 2.20 eV^20^. Remarkably, for group II samples the YL1 band is not observed. Instead, we can see the new YL3 band, which has a maximum at 2.09 eV (Fig. 4). These YL1 and YL3 bands have very distinctive characteristics, which will be analyzed below.

### Shapes of the YL1 and YL3 bands

**Figure 5:**
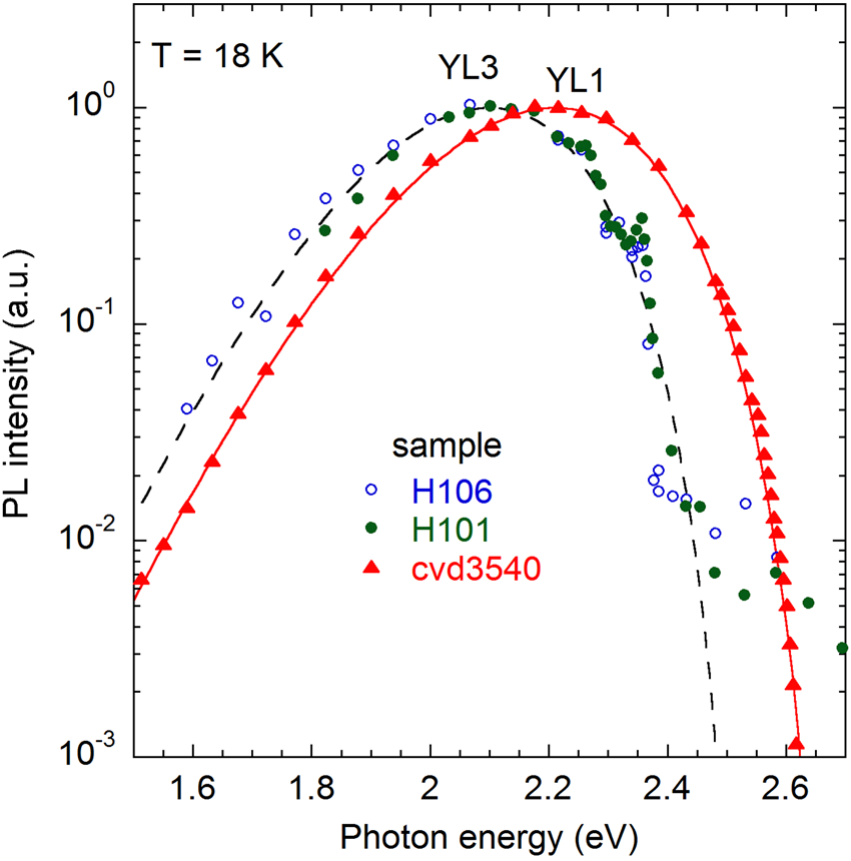
Normalized TRPL spectra from GaN samples at *T* = 18 K. The time delay is 10^−3^ s for samples H101 and H106 (group II) and 10^−6^ s for sample cvd3540 (MOCVD-grown Si-doped GaN). The lines are calculated using Eq. (2) with the following parameters: *S*_*e*_ = 7.4, *E*_0_^*^ = 2.673 eV, $\hslash {\omega }_{{\rm{\max }}}=2.213$ eV (for the YL1 band) and *S*_*e*_ = 7.0, *E*_0_^*^ = 2.52 eV, $\hslash {\omega }_{{\rm{\max }}}=2.09$ eV (for the YL3 band).

In the SSPL spectra, the broad band in the range of photon energies between 1.5 and 2.5 eV always appears as a single band for samples of group II. However, the evolution of the PL spectrum in TRPL measurements clearly shows that the broad band is composed of two bands: fast RL3 and slow YL3 (Fig. 4). Figure 5 compares shapes of the normalized YL1 and YL3 bands obtained from TRPL measurements. The shapes are fitted using Eq. (2) with the fitting parameters indicated in the figure caption. For clarity, the YL1 band spectrum is shown for a MOCVD-grown GaN sample, where the RL1 band is absent. A small correction was made to the $\hslash {\omega }_{{\rm{\max }}}$ and ${E}_{0}^{\ast }$ parameters of the YL1 band to account for a strain-related blue shift (13 meV) for the MOCVD GaN sample. Note that the same YL1 band with a maximum at 2.20 eV and the ZPL at 2.57 eV is detected in GaN samples of group I grown by HVPE and GaN samples grown by MOCVD^18^.

In the fits of the band shapes, the parameters $\hslash {\omega }_{{\rm{\max }}}$ and *S*_*e*_ can be found with high and low precision, respectively. The parameter *S*_*e*_ affects mostly the band width *W* (a larger *S*_*e*_ correlates with a smaller *W*) and its asymmetry (the band shape approaches a Gaussian curve with increasing *S*_*e*_). Note that an increase in *S*_*e*_ can be partly compensated by a small increase in ${E}_{0}^{\ast }$. For example, the shape of the YL3 band at *T* = 18 K can be described with Eq. (2) (within experimental error) where *S*_*e*_ = 7 ± 1, $\hslash {\omega }_{{\rm{\max }}}$ = 2.090 ± 0.005 eV, and ${E}_{0}^{\ast }=2.52\pm 0.02$ eV. The parameter *E*_0_ does not match exactly the experimentally found ZPL because of the model simplicity. We emphasize that interpretation of these parameters is beyond the scope of this work, and Eq. (2) is used here to fit the experimental data and to resolve distinct PL bands.

**Figure 6:**
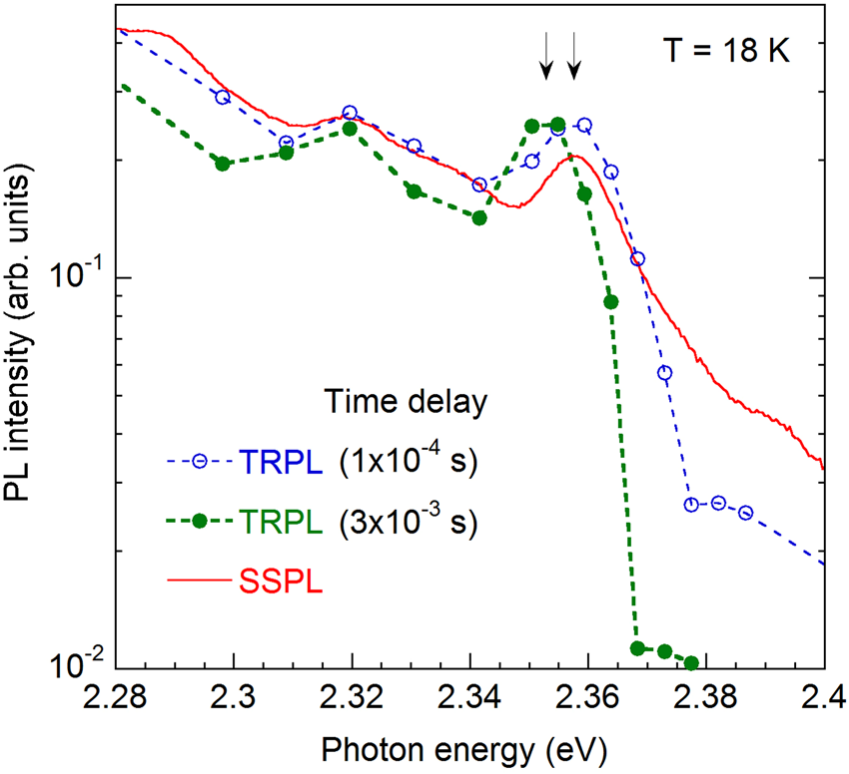
Time evolution of the PL spectrum at the high-energy side of the YL3 band. Sample H106. Positions of the ZPL at two time delays in TRPL are shown with arrows. The solid line shows the SSPL spectrum excited with *P*_*exc*_ = 0.007 W/cm^2^.

The YL3 band has a maximum at 2.09 eV and the ZPL at 2.36 eV. The intensity of the ZPL is 0.2–0.3 of the intensity of the YL3 band maximum. For the YL1 band, these parameters are 2.20 eV, 2.57 eV, and 0.01, respectively^18^. The fine structure of the YL3 band observed in TRPL measurements (Fig. 6) is identical to that of the red band in the SSPL spectra reported in ref.^20^. This observation indicates that the red band in SSPL spectra from group II samples in fact consists of two bands: the featureless fast RL3 band at 1.8 eV and the slow YL3 band with a maximum at 2.09 eV and ZPL at 2.36 eV.

## Discussion

### Fingerprints of the YL1 and YL3 bands

**Figure 7:**
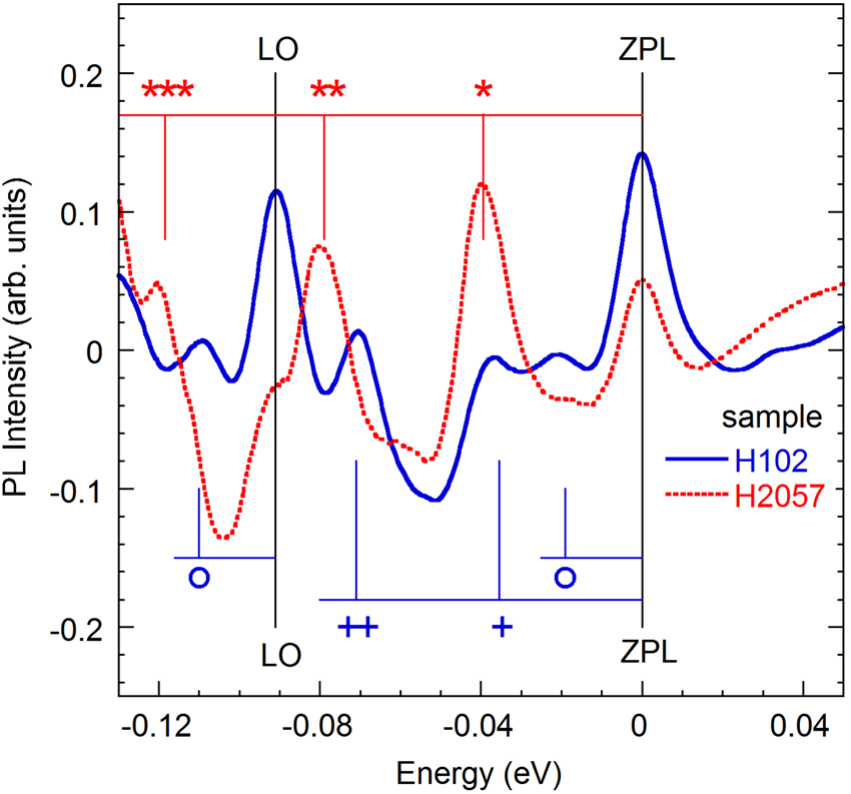
Fine structure of the SSPL spectra from two samples (H102 is from group II and H2057 is from group I). The smooth components for the YL1 and YL3 bands calculated using Eq. (2) with parameters given in the caption to Fig. 5 are subtracted. The spectra are shifted so that the ZPLs are observed at 0. The LO phonon replica at −91 meV is labeled LO. The pseudo-local phonon mode replicas for the YL1 band at energies multiple of 39.5 meV are indicated with stars. The pseudo-local modes for the YL3 band are indicated with circles (19 meV) and crosses (multiple of 36 meV).

Figure 7 compares the fine structure of the YL1 and YL3 bands obtained after subtracting the smooth components which are calculated using Eq. (2). As discussed in ref.^18^, the fine structure of the YL1 band is formed by a superposition of two types of phonon replicas: a pseudo-local mode with a phonon energy of 39.5 meV and an LO lattice mode with a phonon energy of 91 meV. As for the YL3 band, in addition to the LO lattice mode, the superposition includes two pseudo-local modes with phonon energies of 19 and 36 meV. The fine structure of the YL3 band in the TRPL spectrum is less resolved due to technical difficulties (very weak signal at time delays when the YL3 band dominates and insufficient density of experimental data points). However, the ZPL and the first phonon replica (at about 36 meV from the ZPL) could be clearly resolved in Fig. 6.

Equation (2) assumes the existence of one (effective) phonon mode and cannot explain the fine structure of the YL3 band. In the case of three phonon modes, the shape of the PL band can be simulated with a superposition of multiple lines (including the ZPL and all phonon replicas), which have the same shape but different intensities^18,33^. The intensity of each line can be found as^34^:

3 $${W}_{kmn}={W}_{000}\frac{{S}_{1}^{k}}{k!}\frac{{S}_{2}^{m}}{m!}\frac{{S}_{3}^{n}}{n!}.$$

**Figure 8:**
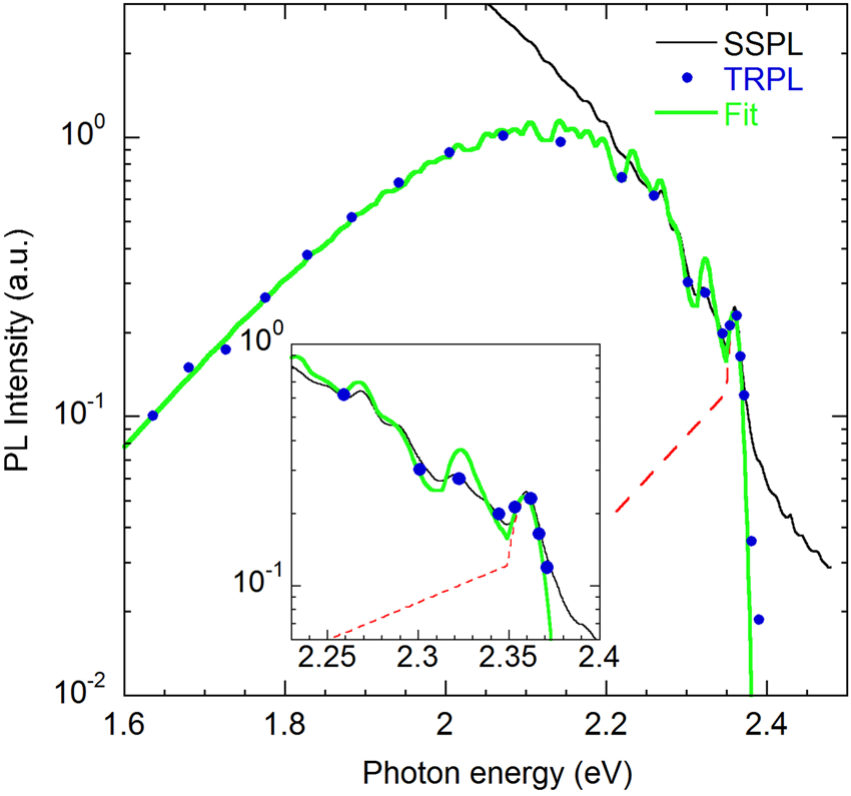
PL spectra of the YL3 band at *T* = 18 K. GaN sample H102 (group II). The symbols show the normalized TRPL spectrum, and the thin solid line shows the SSPL spectrum excited with *P*_*exc*_ = 0.007 W/cm^2^. The thick light green line is calculated using Eq. (3) with parameters *S*_1_ = 0.25, *S*_2_ = 1.0, *S*_3_ = 1.65, and *W*_000_ = 0.23. The dashed line is a simulated shape of the ZPL.

Here, *k*, *m*, and *n* are the number of emitted phonons with energy *ħ*Ω_1_, *ħ*Ω_2_, and *ħ*Ω_3_, respectively; *S*_1_, *S*_2_, and *S*_3_ are the Huang-Rhys factors describing the coupling with these phonon modes, and ${W}_{000}$ is the ZPL intensity. The phonon replicas corresponding to the emission of *k* + *m* + *n* phonons create peaks in the PL spectrum that are shifted to lower energies from the ZPL by $k\hslash {{\rm{\Omega }}}_{1}+m\hslash {{\rm{\Omega }}}_{2}+n\hslash {{\rm{\Omega }}}_{3}$. The YL3 band shape simulated using Eq. (3) with *S*_1_ = 0.25 (*ħ*Ω_1_ = 19 meV), *S*_2_ = 1.0 (*ħ*Ω_1_ = 36 meV), and *S*_3_ = 1.65 (*ħ*Ω_3_ = 91 meV) is shown in Fig. 8 in comparison with the experimental data. The shape of the phonon replicas is assumed to be the same as that of the ZPL. The latter is simulated as consisting of a relatively sharp line and a shoulder at its low-energy side (the dashed line in Fig. 8). This shape is similar to the shape of the UVL band ZPL, and it can be explained by the interaction with acoustic phonons.

The above parameters of the YL3 band fine structure can be compared with those for the YL1 band^18^ and the Zn_Ga_-related BL1 band^33^. The fine structure of the YL1 band is formed by a superposition of the ZPL at 2.57 eV (*W*_000_ = 0.01) and two types of phonon replicas: the pseudo-local phonon mode with *S*_1_ = 4 (*ħ*Ω_1_ = 39.5 meV) and lattice LO mode with *S*_2_ = 2 (*ħ*Ω_2_ = 91.5 meV). The fine structure of the BL1 band can be simulated with the ZPL at 3.10 eV (*W*_000_ = 0.25) and two types of phonon replicas: the pseudo-local phonon mode with *S*_1_ = 1.5 (*ħ*Ω_1_ = 36 meV) and lattice LO mode with *S*_2_ = 2 (*ħ*Ω_2_ = 91.5 meV).

The YL1 and YL3 bands share some common properties, while they have different quantitative characteristics. At low temperatures (below 40 K), the decay of both the YL1 and YL3 bands is nonexponential after a laser pulse. Such decay is typical for DAP transitions involving a shallow donor and a deep defect (usually an acceptor) in GaN^3^. In agreement with this assignment, the ZPL of the YL3 band moves to lower photon energies as time passes after the excitation pulse (Fig. 6). This is because close pairs have a shorter lifetime than more distant pairs and they contribute to the spectrum at a higher photon energy due to stronger Coulomb interaction^25^. At temperatures above 50 K, the PL decays become nearly exponential (Fig. 2), which indicates that electron transitions from the conduction band to the same deep defect (e-A transitions) become dominant. At these temperatures, the DAP-related ZPL and its phonon replicas disappear, and e-A peaks shift to higher energies by about 18 meV emerge, both for the YL1 and YL3 bands^18,20^. The PL lifetimes of the YL1 and YL3 bands decrease inversely proportional to the concentration of free electrons, *n*_0_. This observation also confirms that the electron transitions originate from the conduction band at *T* > 50 K for both bands.

The identification of the transition type and the observation of the ZPL allows for the precise determination of the thermodynamic charge transition levels for defects responsible for the two yellow bands. The YL1 defect has its transition level at 0.916 ± 0.003 eV^18^, whereas the YL3 defect has its transition level at 1.130 ± 0.002 eV above the valence band^20^. From analysis of the PL lifetimes and temperature-dependent Hall effect measurements, we have found the electron-capture coefficients: *C*_*nA*_ = (2.0 ± 0.5) × 10^−13^ cm^3^/s for the YL3 band and *C*_*nA*_ = (1.1 ± 0.2) × 10^−13^ cm^3^/s for the YL1 band. For a set of 11 samples where the YL3 and UVL bands were detected, the ratio of their lifetimes was found to be 18.3 ± 1.7 at 100 K, well outside the statistical range of 29.8 ± 1.2 for the YL1/UVL lifetimes ratio for 10 of the group I samples^23^.

### On the origin of defect-related luminescence bands in undoped GaN

From a large number of GaN samples grown by different techniques, we could not find yellow bands other than the YL1 and YL3 bands, which are investigated in detail here. The new YL3 band is unlikely to be associated with carbon, because it appears only in HVPE GaN with a low concentration of carbon (down to 10^15^ cm^−3^ [ref.^12^]). It is also unlikely to be caused by the gallium vacancy or V_Ga_-related complexes, as will be explained in the next section. Our preliminary analysis of a large amount of experimental data from HVPE GaN samples indicates that the YL3 and RL3 bands may be caused by the same defect. However, currently, we do not have sufficient data to provide convincing evidence and identify this defect.

In contrast to the YL3 band, many experimental factors point to a relationship between the YL1 band and carbon in GaN^3,6,18^. The YL1 band is weak in HVPE GaN where the concentration of carbon is about 10^16^ cm^−3^ or lower. It is very strong in undoped or Si-doped GaN grown by MOCVD, where the concentration of carbon is much higher. We assume that the ubiquitous yellow band observed by many researchers in GaN is the C-related YL1 band. Carbon-free defects, such as the V_Ga_ or V_Ga_-containing complexes should be excluded from candidates for the YL1 band, because they cannot have exactly the same fingerprints (ZPL, band shape, electron- and hole-capture coefficients) as the carbon-associated defect.

According to recent first-principles calculations, the C_N_ acceptor^9^ and the C_N_O_N_ complex^11^ are the best candidates for the YL1 center. However, it would be incorrect to conclude that both C_N_ and C_N_O_N_ defects contribute to the yellow band. Indeed, we observe only one C-related yellow band (YL1) with reproducible position, shape, precisely determined ZPL, and phonon structure in a variety of GaN samples (undoped, C-doped, Si-doped, and Fe-doped) grown by different techniques (MOCVD, HVPE, and MBE). According to first-principles calculations, there should be a significant difference (0.10–0.28 eV) between the energy levels of the C_N_ and C_N_O_N_ defects^10,12^. Below, we provide some arguments that may help to choose between the two models.

The C_N_ defect is expected to have two thermodynamic transition levels: the −/0 level at 0.78–1.04 eV and the 0/+ level at 0.35–0.48 eV above the valence band maximum^10,12,35^. Therefore, two PL bands (the “primary” band is related to transitions via the −/0 level and the “secondary” is related to the 0/+ level) are expected to be observed for different experimental conditions. Transitions via the −/0 level agree with the ionization energy of the YL1 defect (0.916 eV). However, with increasing excitation intensity, when the YL1 band saturates due to the saturation of the YL1 defects with photogenerated holes, another PL band is expected to emerge. This secondary band should correspond to electron transitions from the conduction band to the 0/+ level of the C_N_ defect. Its maximum is expected at higher photon energies^10,12,35^. The GL1 band with a maximum at 2.40 eV was earlier proposed as a suitable candidate for this secondary band^12,32^. Namely, a yellow band with a maximum at 2.10 eV and the GL1 (2.40 eV) band were attributed to transitions via the −/0 and 0/+ levels, respectively, of the C_N_ defect in ref.^12^, because of the apparent correlation between these two bands in sample RS280. However, after the analysis of PL from a large number of GaN samples, we found no correlation between the YL1 and GL1 bands (as well as between the YL3 and GL1 bands). Detailed analysis of PL from sample RS280 indicates that the yellow band with a maximum at 2.10 eV is the YL3 band with its characteristic ZPL and fine structure.

The secondary band, which may be associated with the YL1 defect when it captures two holes, has not been found. The ratio between the quantum efficiencies of the YL1 band before saturation and the unresolved PL background in the green-blue region of the spectrum in samples with a very low background signal in this spectral region is about 10^3^ (see, for example, Fig. 2 in ref.^12^). Then, to explain the absence of the secondary PL band for the YL1 center (assuming that it is the C_N_ defect), one will have to assume that the hole-capture coefficient for the C_N_^0^ defect is smaller than that for the C_N_^−^ by at least 10^3^. Alternatively, the lack of the secondary PL band for the YL1 center could be explained by assuming that the recombination of free electrons with holes bound to the 0/+ level of the C_N_ is nonradiative, or that the 0/+ level is in fact much closer to the valence band, if it exists at all.

The C_N_O_N_ complex is expected to have the 0/+ thermodynamic transition level at 0.68–0.75 eV above the valence band maximum^10,11^. The secondary PL band is not expected for this complex, because the +/2+ level is calculated to be very close to the valence band (at ~0.14 eV)^11^, and, besides, the capture of holes by a positively charged defect is very unlikely. The relatively low binding energy of the C_N_O_N_ complex (about 0.4 eV)^12^ may be a limiting factor in the formation of these defects during growth^10^. However, for temperatures below ~660 K, the complex should be stable^12^. In summary, significant experimental and theoretical evidence suggests that the YL1 band is related to carbon (either the C_N_ or the C_N_O_N_ complex)^3,9–12^, while the exact structure of the defect and the existence of the second transition level remain uncertain.

The identity of the GL1 band, often observed in *n*-type GaN grown by HVPE, deserves a more detailed discussion. The GL1 band is likely to be the secondary band for some defect (the emission band which occurs after the defect captures two holes, not one), because its intensity increases almost as the square of the excitation intensity. However, we could not find any correlation between the quantum efficiencies of the GL1 band and other PL bands at lower photon energies in a large set of HVPE-grown GaN samples. Thus, none of the PL bands observed between 1.5 and 2.3 eV in HVPE GaN (RL1, RL3, YL1, YL3) can be the primary PL band (the emission band which occurs after the defect captures one hole) for the GL1-related defect. One explanation for this would be a very low quantum efficiency of the primary band, much lower than that for the GL1 and other above-mentioned PL bands. However, if the undetected primary band and the GL1 band were caused by transitions via the −/0 and 0/+ levels, respectively, of the same defect, it would be a mystery why the quantum efficiency of the former is much lower than that of the latter. Indeed, holes should be more efficiently captured by the negatively charged defect than by the neutral one. Another explanation for not observing the primary band is that the related transition is nonradiative. In any case, the GL1 band is not likely to be caused by carbon because this band is strong in HVPE-grown GaN where the concentration of C can be as low as 10^15^ cm^−3^ and it is not observed in MOCVD-grown GaN where the concentration of C is 1–3 orders of magnitude higher.

### Comparison with other experimental techniques

It is interesting to compare PL results with data obtained by using complementary techniques. For selected undoped HVPE GaN samples, we have found the concentrations of defects responsible for different PL bands from PL measurements, concentrations of the most common impurities from secondary ion mass spectrometry (SIMS) analysis, and concentrations of free electrons from temperature-dependent Hall effect and TRPL measurements. Additional information was obtained from positron annihilation spectroscopy (PAS) and deep-level transient spectroscopy (DLTS) measurements^36^. The preliminary analysis indicates that group II samples contain lower concentrations of the V_Ga_ than group I samples. This finding indicates that the V_Ga_-related defects are unlikely to be responsible for the YL3 band, yet more PAS data are needed for definite conclusions. None of the impurities (C, O, Si, H, and Cl) showed elevated levels in group II samples, and their concentrations were close to the SIMS detection level. A comparison of the results obtained by PL and DLTS merits a more detailed consideration.

In *n*-type GaN, defects in the lower half of the bandgap can be probed by optical DLTS (ODLTS) or deep-level optical spectroscopy (DLOS). In the ODLTS method, optical pulses are used, instead of electrical pulses, to fill the minority carrier traps in the depletion region of a Schottky diode. Several hole traps were identified in MOCVD and HVPE GaN by ODLTS^37–40^. It appears that the hole trap H1^38,39^ (also called HT1 [ref.^37^] and H(0.85) [ref.^40^]) with an energy level at 0.85–0.95 eV (at 330 K) above the valence band is the same defect as the YL1 center (the energy level at 0.916 eV in the limit of low temperature). This conclusion is based on the similarity of the energy levels and capture cross-sections for the defects observed in PL and ODLTS, as well as on the correlation of the appearance of the yellow band and the H1 trap in GaN samples. Note that the ODLTS measurements provide a rough value of the transition level for the H1 trap (e.g., 0.85 ± 0.05 eV [ref.^40^] and 0.92–0.95. eV [ref.^38^]). In one study, the apparent ionization energy of the H1 trap varied between 0.92 and 1.4 eV when the excitation conditions were changed^41^. This makes it difficult to compare the PL and ODLTS data. The nature of the H1 trap is controversial. According to Auret *et al*.^40^, this defect cannot be the gallium vacancy because it was present in as-grown GaN samples and its concentration decreased after irradiation with 1.8 MeV protons.

Polyakov *et al*.^42^ investigated defects in our group II samples (called group 2 samples in that work) with the ODLTS method. They concluded that the H5 trap with an ionization energy of 1.1 eV (at 370 K) is the dominant hole trap in these samples. It appears that the H5 trap and the YL3 center (with ionization energy of 1.13 eV) are the same defect. However, a similar trap (also called the H5 trap by these authors) with an ionization energy of 1.2 eV is the dominant hole trap in less compensated GaN samples grown by HVPE (similar to our group I samples)^41,42^. Note that defects detected by ODLTS may be nonradiative and may not correspond to any PL band.

In summary, we observed two distinctive PL bands in undoped GaN samples grown by HVPE. The YL1 band has a maximum at 2.20 eV and the ZPL at 2.57 eV at low temperatures. It is caused by a carbon-related defect, which has a thermodynamic charge transition level at 0.916 eV above the valence band maximum. The YL1 band is typical for GaN samples grown by MOCVD and less frequently observed in HVPE-grown GaN samples, because the latter contain much fewer carbon impurities. Another yellow band, the YL3 band, has a maximum at 2.09 eV and the ZPL at 2.36 eV at low temperatures. This band can be resolved only in time-resolved PL measurements and previously was thought to be part of the red band with a maximum at 1.8 eV. The YL3 band is observed in HVPE GaN with a low concentration of free electrons, and its identity remains unknown. Neither the YL1 band nor the YL3 band is related to the same defect as the GL1 band. The GL1 band is the secondary PL band of some defect, which behaves as a giant trap for electrons in GaN. It is often the dominant defect in HVPE-grown GaN, yet its identity is unknown.

## Methods

### Samples

We investigated in detail PL from about 30 undoped GaN samples that can be divided into two groups according to their distinctive PL features. Group I includes ten GaN layers with the typical thickness of about 20 μm grown on sapphire substrates in a horizontal HVPE reactor at temperatures between 950 and 1050 °C. These are conductive *n*-type samples with the room-temperature concentration of free electrons of about 1 × 10^17^ cm^−3^. The samples can be regarded as standard HVPE GaN samples, which typically show the RL1 band at 1.8 eV, the YL1 band at 2.20 eV, the GL1 band at 2.40 eV, the BL1 band at 2.9 eV, and the UVL band with the first peak at 3.27 eV in the PL spectrum^23^. The relative contributions of the above defect-related bands to the PL spectrum are different in different samples and depend on temperature, excitation intensity, and measurement conditions (SSPL or TRPL).

Group II includes four GaN layers with thickness of about 10 μm grown on sapphire in a vertical HVPE reactor at 850 °C. According to capacitance-voltage (C-V) and deep-level transient spectroscopy (DLTS) studies, these samples (called “group 2 samples” in ref.^42^) are highly compensated, with a concentration of uncompensated shallow donors of about 1 × 10^16^ cm^−3^. In agreement with capacitance measurements, the exciton emission intensity in these samples is low and increases super-linearly with excitation intensity, while for group I samples the exciton emission is very strong and increases linearly with excitation intensity. Hall effect measurements show the room-temperature concentration of free electrons between 4 × 10^16^ and 8 × 10^16^ cm^−3^ in these samples. The difference between the data obtained from the C-V and Hall effect measurements can be explained by assuming that different regions were probed by these two techniques. From TRPL measurements and by using a method suggested in ref.^23^, the concentration of free electrons in these samples was estimated to be about 2 × 10^16^ cm^−3^ at 250 K. Details of structural characterization can be found in ref.^42^. In particular, the density of threading dislocations is close to 10^8^ cm^−3^ for samples from both groups I and II.

The dominant PL feature in all the group II samples is the strong RL3 band with a maximum at 1.8 eV and the fine structure on the high-energy side of this band with the ZPL at 2.36 eV^20^. Although the maxima of the RL1 and RL3 bands are observed at the same photon energy (1.8 eV), our TRPL studies indicate that these bands originate from different defects. Several other HVPE GaN samples cannot be attributed uniquely to either group because their PL spectra contain features of both groups. Sample cvd3540, used for comparison with HVPE GaN samples, is a 1.5 μm-thick GaN layer on sapphire substrate, grown by MOCVD and doped with Si ([Si] = 4 × 10^17^ cm^−3^).

### Photoluminescence measurements

The SSPL was excited with an unfocused He-Cd laser (30 mW, 325 nm), dispersed by a 1200 rules/mm grating in a 0.3 m monochromator and detected by the cooled photomultiplier tube R928. Calibrated neutral-density filters were used to attenuate the excitation power density (*P*_exc_) over the range of 10^−7^ −0.2 W/cm^2^. A closed-cycle optical cryostat was used for temperatures between 18 and 320 K. All the samples were measured in identical conditions, and the spectral response of the measurement system was taken into account.

The TRPL was excited with a pulsed nitrogen laser (pulses with duration of 1 ns, repetition frequency of 6 Hz, and photon energy of 3.68 eV), detected with the same photomultiplier tube, and analyzed with a digital oscilloscope (model TDS3052B from Tektronix, Inc.). Results of the present work were obtained by using moderate incident photon flux (*P*_0_ = 5 × 10^21^ cm^−2^ s^−1^ during the pulse). At each photon energy, a PL transient was measured (10,000 data points) by averaging the signal for 1–3 minutes. The signal measured before the pulse arrived was subtracted as a baseline, so that the PL decay could be analyzed over 2–3 orders of magnitude in intensity and time. The TRPL spectra were obtained from these transients at selected time delays. The stability of the signal was checked by measuring the PL decay at a characteristic photon energy (usually at the band maximum) before and after the PL spectrum measurement. The measurements were repeated at different time scales of the oscilloscope and with different slit widths of the monochromator (weaker signal required wider slit width).
